# Chlorido­{2-[(di­methyl­amino)­meth­yl]benzene­seleno­lato-κ^2^ *N*,*Se*}(tri­phenyl­phosphane-κ*P*)palladium(II)

**DOI:** 10.1107/S1600536814010678

**Published:** 2014-05-17

**Authors:** Esther M. Takaluoma, Raija Oilunkaniemi, Risto S. Laitinen

**Affiliations:** a Department of Chemistry, PO Box 3000, FI-90014 University of Oulu, Finland

## Abstract

The asymmetric unit of the title compound, [PdCl(C_9_H_12_NSe)(C_18_H_15_P)], contains two independent mol­ecules. In both cases, the Pd^2+^ cations are coordinated by the Se and N atoms of the chelating bidentate 2-[(di­methyl­amino)­meth­yl]benzene­seleno­late ligand. The chloride ligand lies *trans* to selenium and the tri­phenyl­phosphane ligand is *trans* to nitro­gen. The Pd—Se bond lengths in the two independent coordination environments of Pd are 2.3801 (4) and 2.3852 (4) Å, the Pd—P bond lengths are 2.2562 (8) and 2.2471 (8) Å, the Pd—N bond lengths are 2.172 (2) and 2.158 (2) Å, and the Pd—Cl bond lengths are 2.3816 (8) and 2.3801 (8) Å. The square-planar coordination around one Pd^2+^ cation is less distorted than that around the other.

## Related literature   

For the related structure of a palladium complex with an iodide ligand, see: Chakraborty *et al.* (2011 ▶). For examples of mononuclear platinum complexes, see: Hannu *et al.* (2000 ▶); Hannu-Kuure *et al.* (2003*a* ▶). For mononuclear palladium complexes, see: Risto *et al.* (2007 ▶). For di- and polynuclear palladium complexes, see: Hannu-Kuure *et al.* (2003*b* ▶, 2004 ▶); Wagner *et al.* (2005 ▶).
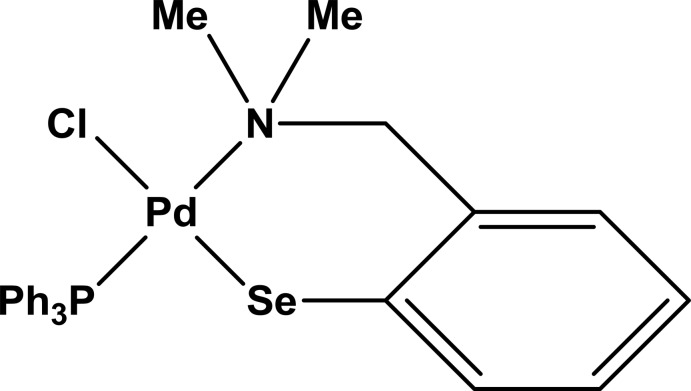


## Experimental   

### 

#### Crystal data  

[PdCl(C_9_H_12_NSe)(C_18_H_15_P)]
*M* *_r_* = 617.28
Triclinic, 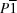

*a* = 13.3528 (3) Å
*b* = 15.0683 (4) Å
*c* = 15.0721 (3) Å
α = 78.857 (1)°
β = 66.385 (1)°
γ = 63.820 (1)°
*V* = 2493.06 (10) Å^3^
*Z* = 4
Mo *K*α radiation
μ = 2.39 mm^−1^
*T* = 120 K
0.10 × 0.10 × 0.08 mm

#### Data collection  

Bruker–Nonius KappaCCD diffractometer
Absorption correction: multi-scan (*SADABS* in *SHELXTL*; Sheldrick, 2008 ▶) *T*_min_ = 0.796, *T*_max_ = 0.832
36674 measured reflections
9759 independent reflections
7312 reflections with *I* > 2σ(*I*)
*R*_int_ = 0.044

#### Refinement  

*R*[*F* ^2^ > 2σ(*F* ^2^)] = 0.031
*wR*(*F* ^2^) = 0.058
*S* = 0.99
9759 reflections
581 parameters
H-atom parameters constrained
Δρ_max_ = 0.65 e Å^−3^
Δρ_min_ = −0.40 e Å^−3^

### 

Data collection: *COLLECT* (Bruker, 2008 ▶); cell refinement: *DENZO-SMN* (Otwinowski & Minor, 1997 ▶); data reduction: *DENZO-SMN*; program(s) used to solve structure: *SHELXS97* (Sheldrick, 2008 ▶); program(s) used to refine structure: *SHELXL97* (Sheldrick, 2008 ▶); molecular graphics: *DIAMOND* (Brandenburg, 2006 ▶); software used to prepare material for publication: *WinGX* (Farrugia, 2012 ▶).

## Supplementary Material

Crystal structure: contains datablock(s) I, global. DOI: 10.1107/S1600536814010678/nk2221sup1.cif

Structure factors: contains datablock(s) I. DOI: 10.1107/S1600536814010678/nk2221Isup2.hkl

CCDC reference: 1002116

Additional supporting information:  crystallographic information; 3D view; checkCIF report

## Figures and Tables

**Fig. 1.**
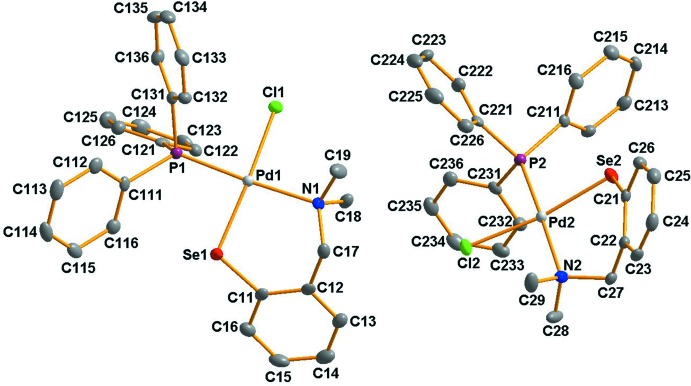
The molecular structure of the title compound indicating the numbering of the atoms. The thermal ellipsoids have been drawn at 50% probability. The hydrogen atoms have been omitted for clarity.

## supplementary crystallographic information

### 1. Comment

The ligand exchange reactions of [MCl_2_(PPh_3_)_2_] (*M* = Pd, Pt) by organoselenolates afford mononuclear metal complexes in the case of platinum, (see for instance, Hannu *et al.*, 2000, Hannu-Kuure *et al.*, 2003*a*), but in case of palladium dinuclear or complexes of even higher nuclearity are generally obtained, as exemplified by Hannu-Kuure *et al.* (2003*b*, 2004) and Wagner *et al.* (2005). Mononuclear palladium complexes can be obtained by using chelating phosphines such as 1,2-bis(diphenylphosphino)ethane (Risto *et al.*, 2007). Organoselenolates containing additional donor atoms can also form stable monomeric palladium complexes. We are interested in the use of the monomeric palladium chalcogenolato complexes as building blocks for the systematic construction of polynuclear metal complexes.

[PdX(C_9_H_12_NSe)(C_18_H_15_P)] (*X* = Br, I) has recently been prepared by the oxidative addition of [2-(*N,N*-dimethylamino)methyl]phenylselenenylbromide or -iodide to [(Ph_3_P)_4_Pd] by Chakraborty *et al.* (2011). They also reported the crystal structure of [PdI(C_9_H_12_NSe)(C_18_H_15_P)]. In this work, the title compound, [PdCl(C_9_H_12_NSe)(C_18_H_15_P)], was formed by the ligand exchange reaction of [PdCl_2_(PPh_3_)_2_] and lithium [2-(*N,N*-dimethylamino)methyl]benzeneselenolate. The ^77^Se NMR spectrum showed, in addition to the chemical shift of the title compound at 258 p.p.m., also a resonance at 422 p.p.m.. It is possible that the selenolate has been oxidized during the reaction and formed a diselenide, the chemical shift of which has also been reported by Chakraborty *et al.* (2011).

The asymmetric unit of the title compound contains two discrete complexes. Both Pd atoms show a distorted square-planar coordination. The bidentate [2-(*N,N*-dimethylamino)methyl]benzeneselenolato chelating ligand is bonded to the metal center *via* selenium and nitrogen donor atoms. The chlorido ligand lies *trans* to selenium and the triphenylphosphine ligand *trans* to nitrogen. While the P—Pd—Cl and Cl—Pd—N angles in both independent complexes in the asymmetric unit are almost identical (P1—Pd1—Cl1 86.56 (3)°, P2—Pd2—Cl2 86.75 (3)° and Cl1—Pd1—N1 90.80 (7)°, Cl2—Pd2—N2 90.79 (7)°), the differences are more prominent in the case of the P—Pd—Se and Se—Pd—N angles (P1—Pd1—Se1 90.14 (2)°, P2—Pd2—Se2 88.69 (2)° and Se1—Pd1—N1 92.62 (6)°, Se2—Pd2—N2 94.53 (7)°). The sum of the bond angles around Pd1 is 360.12° and around Pd2 360.76°. However, the computation of the least-squares planes of the square-planar coordination environments involving Pd1 and Pd2 indicates that in both cases the atoms deviate from planarity. The distortion is more prominent for Pd2 than for Pd1.

The bond lengths and angles around the Pd atoms are shown in Table 1. The Pd1—Se1 bond length is 2.3801 (4) Å and Pd2—Se2 2.3852 (4) Å. They are consistent with the Pd—Se bond (2.4218 (3) Å, 295 (2) K) in the iodido analogue (Chakraborty *et al.*, 2011). The Pd—N bond lengths are 2.172 (2) Å and 2.158 (2) Å, the Pd—Cl are lengths 2.3816 (8) Å and 2.3801 (8) Å, and the Pd—P lengths are 2.2562 (8) Å and 2.2471 (8) Å. The Pd—N and Pd—P lengths in the iodido complex are 2.1958 (18) Å and 2.2429 (5) Å, respectively (Chakraborty *et al.*, 2011).

### 2. Experimental

Freshly prepared diethyl ether solution of lithium[2-(*N,N*-dimethylamino)methyl]benzeneselenolate (2 ml; 0.089 mmol/ml) was added to [PdCl_2_(PPh_3_)_2_] (0.051 g, 0.073 mmol) in 4 ml of THF in an 10 mm NMR tube under an argon atmosphere. The solution immediately turned red and the ^31^P and ^77^Se NMR spectra were recorded. Slow evaporation of the solution gave a small crop of red crystals of [PdCl(C_9_H_12_NSe)(C_18_H_15_P)]. NMR data of the title compound: ^77^Se NMR 258 p.p.m., ^31^P NMR 32.2 p.p.m..

### 3. Refinement

H atoms were positioned geometrically and refined using a riding model with C—H = 0.95 Å and with *U*_iso_(H) = 1.2 *U*_eq_(C) and 0.98 Å and *U*_iso_(H) = 1.2 *U*_eq_(C) for the aryl and methyl H atoms, respectively.

### Crystal data

**Table:**

[PdCl(C_9_H_12_NSe)(C_18_H_15_P)]	*Z* = 4
*M**_r_* = 617.28	*F*(000) = 1232
Triclinic, *P*1	*D*_x_ = 1.645 Mg m^−^^3^
Hall symbol: -P 1	Mo *K*α radiation, λ = 0.71073 Å
*a* = 13.3528 (3) Å	Cell parameters from 7312 reflections
*b* = 15.0683 (4) Å	θ = 1.5–26.0°
*c* = 15.0721 (3) Å	µ = 2.39 mm^−^^1^
α = 78.857 (1)°	*T* = 120 K
β = 66.385 (1)°	Block, red
γ = 63.820 (1)°	0.1 × 0.1 × 0.08 mm
*V* = 2493.06 (10) Å^3^	

### Data collection

**Table:**

Bruker–Nonius KappaCCD diffractometer	9759 independent reflections
Radiation source: fine-focus sealed tube	7312 reflections with *I* > 2σ(*I*)
Graphite monochromator	*R*_int_ = 0.044
φ scans, and ω scans with κ offsets	θ_max_ = 26.0°, θ_min_ = 1.5°
Absorption correction: multi-scan (*SADABS* in *SHELXTL*; Sheldrick, 2008)	*h* = −16→15
*T*_min_ = 0.796, *T*_max_ = 0.832	*k* = −18→18
36674 measured reflections	*l* = −18→18

### Refinement

**Table:**

Refinement on *F*^2^	Primary atom site location: structure-invariant direct methods
Least-squares matrix: full	Secondary atom site location: difference Fourier map
*R*[*F*^2^ > 2σ(*F*^2^)] = 0.031	Hydrogen site location: inferred from neighbouring sites
*wR*(*F*^2^) = 0.058	H-atom parameters constrained
*S* = 0.99	*w* = 1/[σ^2^(*F*_o_^2^) + (0.0251*P*)^2^] where *P* = (*F*_o_^2^ + 2*F*_c_^2^)/3
9759 reflections	(Δ/σ)_max_ = 0.001
581 parameters	Δρ_max_ = 0.65 e Å^−^^3^
0 restraints	Δρ_min_ = −0.40 e Å^−^^3^

### Special details

**Table:**

Geometry. All e.s.d.'s (except the e.s.d. in the dihedral angle between two l.s. planes) are estimated using the full covariance matrix. The cell e.s.d.'s are taken into account individually in the estimation of e.s.d.'s in distances, angles and torsion angles; correlations between e.s.d.'s in cell parameters are only used when they are defined by crystal symmetry. An approximate (isotropic) treatment of cell e.s.d.'s is used for estimating e.s.d.'s involving l.s. planes.
Refinement. Refinement of *F*^2^ against ALL reflections. The weighted *R*-factor *wR* and goodness of fit *S* are based on *F*^2^, conventional *R*-factors *R* are based on *F*, with *F* set to zero for negative *F*^2^. The threshold expression of *F*^2^ > σ(*F*^2^) is used only for calculating *R*-factors(gt) *etc*. and is not relevant to the choice of reflections for refinement. *R*-factors based on *F*^2^ are statistically about twice as large as those based on *F*, and *R*- factors based on ALL data will be even larger.

### Fractional atomic coordinates and isotropic or equivalent isotropic displacement parameters (Å^2^)

**Table:**

	*x*	*y*	*z*	*U*_iso_*/*U*_eq_	
C11	0.1449 (3)	0.2371 (2)	0.5732 (2)	0.0164 (7)	
C12	0.2527 (3)	0.2295 (2)	0.4999 (2)	0.0164 (7)	
C13	0.3578 (3)	0.1528 (2)	0.5048 (2)	0.0220 (8)	
H13	0.4318	0.1468	0.4548	0.026*	
C14	0.3564 (3)	0.0852 (3)	0.5809 (3)	0.0280 (9)	
H14	0.4292	0.0346	0.5846	0.034*	
C15	0.2487 (3)	0.0916 (3)	0.6518 (2)	0.0270 (8)	
H15	0.2471	0.0441	0.7034	0.032*	
C16	0.1434 (3)	0.1667 (3)	0.6478 (2)	0.0235 (8)	
H16	0.0694	0.1703	0.6963	0.028*	
C17	0.2546 (3)	0.3041 (2)	0.4188 (2)	0.0179 (7)	
H17A	0.3387	0.2939	0.3802	0.022*	
H17B	0.2127	0.3710	0.4464	0.022*	
C18	0.2621 (3)	0.2010 (2)	0.3097 (2)	0.0229 (8)	
H18A	0.2318	0.1997	0.2608	0.034*	
H18B	0.2498	0.1514	0.3605	0.034*	
H18C	0.3477	0.1863	0.2792	0.034*	
C19	0.2155 (3)	0.3743 (3)	0.2741 (2)	0.0270 (9)	
H19A	0.3012	0.3583	0.2413	0.040*	
H19B	0.1753	0.4402	0.3019	0.040*	
H19C	0.1820	0.3735	0.2273	0.040*	
C21	0.9319 (3)	0.2784 (2)	−0.0904 (2)	0.0158 (7)	
C22	0.9714 (3)	0.2729 (2)	−0.0157 (2)	0.0166 (7)	
C23	1.0138 (3)	0.3420 (3)	−0.0142 (2)	0.0216 (8)	
H23	1.0387	0.3398	0.0374	0.026*	
C24	1.0201 (3)	0.4135 (3)	−0.0864 (2)	0.0225 (8)	
H24	1.0509	0.4591	−0.0853	0.027*	
C25	0.9815 (3)	0.4183 (3)	−0.1603 (2)	0.0235 (8)	
H25	0.9867	0.4669	−0.2105	0.028*	
C26	0.9355 (3)	0.3528 (2)	−0.1612 (2)	0.0203 (8)	
H26	0.9058	0.3585	−0.2107	0.024*	
C27	0.9727 (3)	0.1921 (3)	0.0590 (2)	0.0204 (8)	
H27A	1.0165	0.1934	0.0981	0.024*	
H27B	1.0176	0.1280	0.0258	0.024*	
C28	0.8720 (3)	0.1153 (3)	0.1977 (2)	0.0330 (10)	
H28A	0.9178	0.1220	0.2313	0.050*	
H28B	0.9170	0.0521	0.1643	0.050*	
H28C	0.7950	0.1173	0.2450	0.050*	
C29	0.7846 (3)	0.2930 (3)	0.1772 (2)	0.0301 (9)	
H29A	0.7102	0.2933	0.2278	0.045*	
H29B	0.7661	0.3475	0.1309	0.045*	
H29C	0.8334	0.3011	0.2067	0.045*	
C111	−0.2885 (3)	0.4327 (2)	0.5935 (2)	0.0146 (7)	
C112	−0.3750 (3)	0.5292 (2)	0.6058 (2)	0.0192 (8)	
H112	−0.3854	0.5696	0.5507	0.023*	
C113	−0.4464 (3)	0.5669 (3)	0.6979 (2)	0.0233 (8)	
H113	−0.5050	0.6330	0.7053	0.028*	
C114	−0.4328 (3)	0.5097 (3)	0.7779 (2)	0.0238 (8)	
H114	−0.4816	0.5361	0.8408	0.029*	
C115	−0.3473 (3)	0.4126 (3)	0.7673 (2)	0.0219 (8)	
H115	−0.3381	0.3726	0.8230	0.026*	
C116	−0.2757 (3)	0.3745 (2)	0.6758 (2)	0.0191 (7)	
H116	−0.2174	0.3082	0.6688	0.023*	
C121	−0.2355 (3)	0.2802 (2)	0.4760 (2)	0.0147 (7)	
C122	−0.1492 (3)	0.1870 (2)	0.4420 (2)	0.0174 (7)	
H122	−0.0671	0.1758	0.4160	0.021*	
C123	−0.1819 (3)	0.1108 (2)	0.4459 (2)	0.0222 (8)	
H123	−0.1225	0.0472	0.4231	0.027*	
C124	−0.3016 (3)	0.1273 (3)	0.4829 (2)	0.0260 (8)	
H124	−0.3243	0.0753	0.4846	0.031*	
C125	−0.3882 (3)	0.2190 (3)	0.5175 (2)	0.0260 (8)	
H125	−0.4702	0.2301	0.5431	0.031*	
C126	−0.3553 (3)	0.2948 (3)	0.5148 (2)	0.0200 (8)	
H126	−0.4150	0.3576	0.5396	0.024*	
C131	−0.2533 (3)	0.4777 (2)	0.3930 (2)	0.0143 (7)	
C132	−0.2229 (3)	0.5584 (2)	0.3751 (2)	0.0178 (7)	
H132	−0.1716	0.5601	0.4032	0.021*	
C133	−0.2671 (3)	0.6358 (3)	0.3165 (2)	0.0222 (8)	
H133	−0.2480	0.6917	0.3057	0.027*	
C134	−0.3391 (3)	0.6323 (3)	0.2736 (2)	0.0228 (8)	
H134	−0.3688	0.6853	0.2326	0.027*	
C135	−0.3678 (3)	0.5526 (3)	0.2899 (2)	0.0241 (8)	
H135	−0.4171	0.5504	0.2598	0.029*	
C136	−0.3258 (3)	0.4747 (2)	0.3500 (2)	0.0186 (7)	
H136	−0.3467	0.4198	0.3614	0.022*	
C211	0.7140 (3)	0.0859 (2)	−0.1210 (2)	0.0146 (7)	
C212	0.7818 (3)	−0.0143 (2)	−0.1386 (2)	0.0195 (8)	
H212	0.7871	−0.0598	−0.0857	0.023*	
C213	0.8420 (3)	−0.0487 (3)	−0.2323 (2)	0.0213 (8)	
H213	0.8891	−0.1174	−0.2435	0.026*	
C214	0.8334 (3)	0.0167 (3)	−0.3097 (2)	0.0203 (8)	
H214	0.8748	−0.0067	−0.3741	0.024*	
C215	0.7646 (3)	0.1163 (3)	−0.2930 (2)	0.0229 (8)	
H215	0.7576	0.1612	−0.3461	0.028*	
C216	0.7056 (3)	0.1514 (2)	−0.1993 (2)	0.0198 (8)	
H216	0.6593	0.2202	−0.1886	0.024*	
C221	0.5052 (3)	0.2371 (2)	0.0011 (2)	0.0165 (7)	
C222	0.4255 (3)	0.2250 (3)	−0.0284 (2)	0.0236 (8)	
H222	0.4429	0.1620	−0.0481	0.028*	
C223	0.3209 (3)	0.3047 (3)	−0.0290 (2)	0.0295 (9)	
H223	0.2650	0.2956	−0.0464	0.035*	
C224	0.2978 (3)	0.3969 (3)	−0.0045 (2)	0.0337 (10)	
H224	0.2267	0.4516	−0.0062	0.040*	
C225	0.3771 (3)	0.4106 (3)	0.0227 (2)	0.0313 (9)	
H225	0.3611	0.4745	0.0392	0.038*	
C226	0.4803 (3)	0.3304 (2)	0.0255 (2)	0.0227 (8)	
H226	0.5348	0.3396	0.0445	0.027*	
C231	0.6093 (3)	0.0362 (2)	0.0808 (2)	0.0156 (7)	
C232	0.7025 (3)	−0.0437 (2)	0.1021 (2)	0.0212 (8)	
H232	0.7822	−0.0488	0.0694	0.025*	
C233	0.6809 (3)	−0.1148 (3)	0.1694 (2)	0.0249 (8)	
H233	0.7454	−0.1700	0.1814	0.030*	
C234	0.5645 (3)	−0.1058 (3)	0.2197 (2)	0.0258 (8)	
H234	0.5488	−0.1535	0.2682	0.031*	
C235	0.4721 (3)	−0.0279 (3)	0.1993 (2)	0.0246 (8)	
H235	0.3924	−0.0222	0.2337	0.029*	
C236	0.4932 (3)	0.0425 (2)	0.1294 (2)	0.0195 (7)	
H236	0.4287	0.0950	0.1145	0.023*	
N1	0.1976 (2)	0.30004 (19)	0.35255 (17)	0.0147 (6)	
N2	0.8515 (2)	0.1977 (2)	0.12601 (18)	0.0190 (6)	
P1	−0.19223 (7)	0.38005 (6)	0.47282 (6)	0.01370 (18)	
P2	0.64356 (7)	0.13454 (6)	0.00150 (6)	0.01466 (18)	
Cl1	0.00473 (7)	0.31983 (6)	0.26248 (5)	0.01847 (18)	
Cl2	0.59361 (7)	0.18539 (6)	0.21434 (5)	0.02087 (19)	
Se1	−0.00116 (3)	0.34635 (3)	0.57421 (2)	0.02091 (9)	
Se2	0.87847 (3)	0.18355 (3)	−0.10148 (2)	0.02016 (8)	
Pd1	0.00710 (2)	0.335783 (18)	0.415702 (17)	0.01296 (6)	
Pd2	0.74755 (2)	0.175273 (18)	0.060536 (17)	0.01408 (6)	

### Atomic displacement parameters (Å^2^)

**Table:**

	*U*^11^	*U*^22^	*U*^33^	*U*^12^	*U*^13^	*U*^23^
C11	0.0159 (17)	0.0198 (19)	0.0172 (17)	−0.0060 (15)	−0.0080 (15)	−0.0068 (15)
C12	0.0178 (17)	0.0214 (19)	0.0167 (17)	−0.0086 (15)	−0.0094 (15)	−0.0062 (15)
C13	0.0183 (18)	0.025 (2)	0.0248 (19)	−0.0054 (16)	−0.0098 (16)	−0.0074 (16)
C14	0.028 (2)	0.024 (2)	0.032 (2)	−0.0002 (17)	−0.0180 (18)	−0.0084 (17)
C15	0.041 (2)	0.024 (2)	0.0214 (19)	−0.0131 (18)	−0.0179 (18)	0.0029 (16)
C16	0.0261 (19)	0.031 (2)	0.0172 (18)	−0.0149 (17)	−0.0081 (16)	0.0001 (16)
C17	0.0152 (17)	0.024 (2)	0.0187 (18)	−0.0103 (15)	−0.0035 (15)	−0.0076 (15)
C18	0.0153 (17)	0.029 (2)	0.0243 (19)	−0.0041 (16)	−0.0086 (15)	−0.0100 (16)
C19	0.029 (2)	0.039 (2)	0.0212 (19)	−0.0238 (18)	−0.0107 (17)	0.0099 (17)
C21	0.0094 (15)	0.0208 (19)	0.0161 (17)	−0.0059 (14)	−0.0022 (14)	−0.0038 (14)
C22	0.0100 (16)	0.0215 (19)	0.0143 (17)	−0.0027 (14)	−0.0033 (14)	−0.0033 (14)
C23	0.0132 (17)	0.037 (2)	0.0186 (18)	−0.0087 (16)	−0.0074 (15)	−0.0080 (16)
C24	0.0144 (17)	0.027 (2)	0.027 (2)	−0.0096 (16)	−0.0025 (16)	−0.0098 (17)
C25	0.0199 (18)	0.025 (2)	0.0208 (19)	−0.0079 (16)	−0.0051 (16)	0.0003 (16)
C26	0.0179 (17)	0.029 (2)	0.0146 (18)	−0.0093 (16)	−0.0062 (15)	−0.0006 (15)
C27	0.0108 (16)	0.032 (2)	0.0183 (18)	−0.0068 (15)	−0.0058 (14)	−0.0038 (16)
C28	0.0223 (19)	0.060 (3)	0.024 (2)	−0.0245 (19)	−0.0142 (17)	0.0173 (19)
C29	0.0189 (18)	0.048 (3)	0.029 (2)	−0.0178 (18)	−0.0016 (17)	−0.0186 (19)
C111	0.0108 (16)	0.0181 (18)	0.0164 (17)	−0.0092 (14)	−0.0012 (14)	−0.0036 (14)
C112	0.0151 (17)	0.022 (2)	0.0256 (19)	−0.0114 (15)	−0.0066 (15)	−0.0017 (16)
C113	0.0178 (18)	0.0162 (19)	0.034 (2)	−0.0076 (15)	−0.0020 (16)	−0.0110 (16)
C114	0.0224 (19)	0.030 (2)	0.0216 (19)	−0.0173 (17)	0.0020 (16)	−0.0126 (17)
C115	0.0247 (19)	0.033 (2)	0.0164 (18)	−0.0208 (17)	−0.0053 (16)	0.0008 (16)
C116	0.0172 (17)	0.0212 (19)	0.0223 (19)	−0.0103 (15)	−0.0070 (15)	−0.0018 (15)
C121	0.0197 (17)	0.0166 (18)	0.0119 (16)	−0.0098 (15)	−0.0096 (14)	0.0059 (14)
C122	0.0180 (17)	0.0203 (19)	0.0133 (17)	−0.0078 (15)	−0.0051 (14)	0.0002 (14)
C123	0.032 (2)	0.0174 (19)	0.0165 (18)	−0.0094 (16)	−0.0065 (16)	−0.0049 (15)
C124	0.040 (2)	0.027 (2)	0.024 (2)	−0.0241 (19)	−0.0130 (18)	0.0017 (17)
C125	0.0245 (19)	0.034 (2)	0.027 (2)	−0.0195 (18)	−0.0089 (17)	0.0005 (17)
C126	0.0179 (18)	0.022 (2)	0.0199 (18)	−0.0068 (15)	−0.0077 (15)	−0.0010 (15)
C131	0.0114 (16)	0.0131 (18)	0.0130 (17)	−0.0006 (14)	−0.0032 (14)	−0.0021 (14)
C132	0.0174 (17)	0.022 (2)	0.0151 (17)	−0.0086 (15)	−0.0070 (15)	0.0010 (15)
C133	0.0261 (19)	0.022 (2)	0.0163 (18)	−0.0127 (16)	−0.0025 (16)	0.0008 (15)
C134	0.0246 (19)	0.023 (2)	0.0169 (18)	−0.0066 (16)	−0.0093 (16)	0.0031 (15)
C135	0.0249 (19)	0.030 (2)	0.0226 (19)	−0.0097 (17)	−0.0154 (16)	0.0010 (16)
C136	0.0152 (17)	0.0168 (19)	0.0234 (19)	−0.0069 (14)	−0.0051 (15)	−0.0025 (15)
C211	0.0117 (16)	0.0195 (19)	0.0138 (17)	−0.0085 (14)	−0.0026 (14)	−0.0016 (14)
C212	0.0176 (17)	0.022 (2)	0.0196 (18)	−0.0043 (15)	−0.0106 (15)	−0.0026 (15)
C213	0.0172 (17)	0.021 (2)	0.027 (2)	−0.0041 (15)	−0.0100 (16)	−0.0070 (16)
C214	0.0158 (17)	0.031 (2)	0.0145 (18)	−0.0111 (16)	−0.0020 (15)	−0.0059 (16)
C215	0.0257 (19)	0.028 (2)	0.0177 (18)	−0.0148 (17)	−0.0067 (16)	0.0006 (16)
C216	0.0216 (18)	0.0163 (19)	0.0206 (19)	−0.0078 (15)	−0.0054 (15)	−0.0026 (15)
C221	0.0169 (17)	0.0217 (19)	0.0079 (16)	−0.0065 (15)	−0.0038 (14)	0.0014 (14)
C222	0.0185 (18)	0.032 (2)	0.0182 (18)	−0.0051 (16)	−0.0064 (15)	−0.0085 (16)
C223	0.0161 (18)	0.048 (3)	0.021 (2)	−0.0057 (18)	−0.0103 (16)	−0.0032 (18)
C224	0.025 (2)	0.038 (3)	0.019 (2)	0.0052 (18)	−0.0094 (17)	−0.0032 (18)
C225	0.037 (2)	0.025 (2)	0.020 (2)	−0.0004 (18)	−0.0109 (18)	−0.0052 (17)
C226	0.0244 (19)	0.025 (2)	0.0177 (18)	−0.0084 (16)	−0.0084 (16)	−0.0006 (16)
C231	0.0204 (18)	0.0168 (18)	0.0111 (16)	−0.0090 (15)	−0.0046 (14)	−0.0023 (14)
C232	0.0197 (18)	0.028 (2)	0.0166 (18)	−0.0102 (16)	−0.0061 (15)	−0.0002 (16)
C233	0.031 (2)	0.023 (2)	0.0191 (19)	−0.0068 (17)	−0.0103 (17)	−0.0027 (16)
C234	0.039 (2)	0.021 (2)	0.0196 (19)	−0.0161 (18)	−0.0081 (17)	0.0008 (16)
C235	0.026 (2)	0.032 (2)	0.0208 (19)	−0.0188 (18)	−0.0019 (16)	−0.0065 (17)
C236	0.0209 (18)	0.0200 (19)	0.0200 (18)	−0.0085 (15)	−0.0072 (15)	−0.0052 (15)
N1	0.0144 (14)	0.0196 (15)	0.0125 (14)	−0.0097 (12)	−0.0042 (11)	0.0005 (12)
N2	0.0173 (14)	0.0288 (17)	0.0142 (14)	−0.0112 (13)	−0.0069 (12)	0.0001 (13)
P1	0.0127 (4)	0.0143 (5)	0.0145 (4)	−0.0053 (4)	−0.0048 (4)	−0.0016 (4)
P2	0.0136 (4)	0.0166 (5)	0.0137 (4)	−0.0053 (4)	−0.0051 (4)	−0.0016 (4)
Cl1	0.0187 (4)	0.0233 (5)	0.0154 (4)	−0.0082 (4)	−0.0076 (3)	−0.0022 (3)
Cl2	0.0169 (4)	0.0301 (5)	0.0160 (4)	−0.0119 (4)	−0.0004 (3)	−0.0076 (4)
Se1	0.01454 (17)	0.0306 (2)	0.01433 (18)	−0.00349 (15)	−0.00578 (14)	−0.00631 (15)
Se2	0.02192 (18)	0.0287 (2)	0.01329 (17)	−0.01509 (16)	−0.00248 (15)	−0.00404 (15)
Pd1	0.01155 (12)	0.01608 (14)	0.01212 (13)	−0.00552 (10)	−0.00456 (10)	−0.00157 (10)
Pd2	0.01134 (12)	0.01862 (15)	0.01210 (13)	−0.00543 (11)	−0.00354 (10)	−0.00296 (10)

### Geometric parameters (Å, º)

**Table:**

C11—C16	1.390 (4)	C123—C124	1.384 (5)
C11—C12	1.391 (4)	C123—H123	0.9500
C11—Se1	1.915 (3)	C124—C125	1.380 (5)
C12—C13	1.388 (4)	C124—H124	0.9500
C12—C17	1.493 (4)	C125—C126	1.382 (4)
C13—C14	1.380 (5)	C125—H125	0.9500
C13—H13	0.9500	C126—H126	0.9500
C14—C15	1.381 (5)	C131—C136	1.384 (4)
C14—H14	0.9500	C131—C132	1.391 (4)
C15—C16	1.377 (5)	C131—P1	1.822 (3)
C15—H15	0.9500	C132—C133	1.379 (4)
C16—H16	0.9500	C132—H132	0.9500
C17—N1	1.501 (4)	C133—C134	1.380 (4)
C17—H17A	0.9900	C133—H133	0.9500
C17—H17B	0.9900	C134—C135	1.366 (5)
C18—N1	1.476 (4)	C134—H134	0.9500
C18—H18A	0.9800	C135—C136	1.388 (4)
C18—H18B	0.9800	C135—H135	0.9500
C18—H18C	0.9800	C136—H136	0.9500
C19—N1	1.485 (4)	C211—C212	1.387 (4)
C19—H19A	0.9800	C211—C216	1.392 (4)
C19—H19B	0.9800	C211—P2	1.831 (3)
C19—H19C	0.9800	C212—C213	1.385 (4)
C21—C26	1.395 (4)	C212—H212	0.9500
C21—C22	1.398 (4)	C213—C214	1.381 (4)
C21—Se2	1.914 (3)	C213—H213	0.9500
C22—C23	1.394 (4)	C214—C215	1.379 (5)
C22—C27	1.489 (4)	C214—H214	0.9500
C23—C24	1.380 (5)	C215—C216	1.387 (4)
C23—H23	0.9500	C215—H215	0.9500
C24—C25	1.381 (4)	C216—H216	0.9500
C24—H24	0.9500	C221—C226	1.383 (4)
C25—C26	1.378 (4)	C221—C222	1.396 (4)
C25—H25	0.9500	C221—P2	1.813 (3)
C26—H26	0.9500	C222—C223	1.385 (4)
C27—N2	1.500 (4)	C222—H222	0.9500
C27—H27A	0.9900	C223—C224	1.376 (5)
C27—H27B	0.9900	C223—H223	0.9500
C28—N2	1.485 (4)	C224—C225	1.380 (5)
C28—H28A	0.9800	C224—H224	0.9500
C28—H28B	0.9800	C225—C226	1.385 (5)
C28—H28C	0.9800	C225—H225	0.9500
C29—N2	1.482 (4)	C226—H226	0.9500
C29—H29A	0.9800	C231—C236	1.392 (4)
C29—H29B	0.9800	C231—C232	1.397 (4)
C29—H29C	0.9800	C231—P2	1.819 (3)
C111—C112	1.390 (4)	C232—C233	1.370 (4)
C111—C116	1.398 (4)	C232—H232	0.9500
C111—P1	1.837 (3)	C233—C234	1.385 (5)
C112—C113	1.387 (4)	C233—H233	0.9500
C112—H112	0.9500	C234—C235	1.372 (5)
C113—C114	1.367 (5)	C234—H234	0.9500
C113—H113	0.9500	C235—C236	1.381 (4)
C114—C115	1.392 (5)	C235—H235	0.9500
C114—H114	0.9500	C236—H236	0.9500
C115—C116	1.383 (4)	N1—Pd1	2.172 (2)
C115—H115	0.9500	N2—Pd2	2.158 (2)
C116—H116	0.9500	P1—Pd1	2.2562 (8)
C121—C126	1.392 (4)	P2—Pd2	2.2471 (8)
C121—C122	1.392 (4)	Cl1—Pd1	2.3816 (8)
C121—P1	1.822 (3)	Cl2—Pd2	2.3801 (8)
C122—C123	1.383 (4)	Se1—Pd1	2.3801 (4)
C122—H122	0.9500	Se2—Pd2	2.3852 (4)
			
C16—C11—C12	119.9 (3)	C136—C131—P1	123.6 (2)
C16—C11—Se1	119.7 (2)	C132—C131—P1	116.8 (2)
C12—C11—Se1	120.4 (2)	C133—C132—C131	120.1 (3)
C13—C12—C11	118.9 (3)	C133—C132—H132	120.0
C13—C12—C17	121.0 (3)	C131—C132—H132	120.0
C11—C12—C17	120.1 (3)	C132—C133—C134	120.0 (3)
C14—C13—C12	121.0 (3)	C132—C133—H133	120.0
C14—C13—H13	119.5	C134—C133—H133	120.0
C12—C13—H13	119.5	C135—C134—C133	120.1 (3)
C13—C14—C15	119.6 (3)	C135—C134—H134	119.9
C13—C14—H14	120.2	C133—C134—H134	119.9
C15—C14—H14	120.2	C134—C135—C136	120.7 (3)
C16—C15—C14	120.2 (3)	C134—C135—H135	119.7
C16—C15—H15	119.9	C136—C135—H135	119.7
C14—C15—H15	119.9	C131—C136—C135	119.5 (3)
C15—C16—C11	120.3 (3)	C131—C136—H136	120.3
C15—C16—H16	119.8	C135—C136—H136	120.3
C11—C16—H16	119.8	C212—C211—C216	118.8 (3)
C12—C17—N1	114.1 (2)	C212—C211—P2	121.7 (2)
C12—C17—H17A	108.7	C216—C211—P2	119.3 (2)
N1—C17—H17A	108.7	C213—C212—C211	120.8 (3)
C12—C17—H17B	108.7	C213—C212—H212	119.6
N1—C17—H17B	108.7	C211—C212—H212	119.6
H17A—C17—H17B	107.6	C214—C213—C212	120.0 (3)
N1—C18—H18A	109.5	C214—C213—H213	120.0
N1—C18—H18B	109.5	C212—C213—H213	120.0
H18A—C18—H18B	109.5	C215—C214—C213	119.7 (3)
N1—C18—H18C	109.5	C215—C214—H214	120.2
H18A—C18—H18C	109.5	C213—C214—H214	120.2
H18B—C18—H18C	109.5	C214—C215—C216	120.5 (3)
N1—C19—H19A	109.5	C214—C215—H215	119.7
N1—C19—H19B	109.5	C216—C215—H215	119.7
H19A—C19—H19B	109.5	C215—C216—C211	120.1 (3)
N1—C19—H19C	109.5	C215—C216—H216	119.9
H19A—C19—H19C	109.5	C211—C216—H216	119.9
H19B—C19—H19C	109.5	C226—C221—C222	118.8 (3)
C26—C21—C22	119.2 (3)	C226—C221—P2	119.9 (2)
C26—C21—Se2	118.7 (2)	C222—C221—P2	121.2 (3)
C22—C21—Se2	122.1 (2)	C223—C222—C221	120.1 (3)
C23—C22—C21	119.1 (3)	C223—C222—H222	119.9
C23—C22—C27	120.4 (3)	C221—C222—H222	119.9
C21—C22—C27	120.5 (3)	C224—C223—C222	120.0 (3)
C24—C23—C22	121.0 (3)	C224—C223—H223	120.0
C24—C23—H23	119.5	C222—C223—H223	120.0
C22—C23—H23	119.5	C223—C224—C225	120.5 (3)
C23—C24—C25	119.7 (3)	C223—C224—H224	119.7
C23—C24—H24	120.2	C225—C224—H224	119.7
C25—C24—H24	120.2	C224—C225—C226	119.4 (4)
C26—C25—C24	120.1 (3)	C224—C225—H225	120.3
C26—C25—H25	119.9	C226—C225—H225	120.3
C24—C25—H25	119.9	C221—C226—C225	121.0 (3)
C25—C26—C21	120.8 (3)	C221—C226—H226	119.5
C25—C26—H26	119.6	C225—C226—H226	119.5
C21—C26—H26	119.6	C236—C231—C232	118.6 (3)
C22—C27—N2	114.7 (3)	C236—C231—P2	122.9 (2)
C22—C27—H27A	108.6	C232—C231—P2	118.1 (2)
N2—C27—H27A	108.6	C233—C232—C231	121.0 (3)
C22—C27—H27B	108.6	C233—C232—H232	119.5
N2—C27—H27B	108.6	C231—C232—H232	119.5
H27A—C27—H27B	107.6	C232—C233—C234	119.7 (3)
N2—C28—H28A	109.5	C232—C233—H233	120.1
N2—C28—H28B	109.5	C234—C233—H233	120.1
H28A—C28—H28B	109.5	C235—C234—C233	119.8 (3)
N2—C28—H28C	109.5	C235—C234—H234	120.1
H28A—C28—H28C	109.5	C233—C234—H234	120.1
H28B—C28—H28C	109.5	C234—C235—C236	120.9 (3)
N2—C29—H29A	109.5	C234—C235—H235	119.6
N2—C29—H29B	109.5	C236—C235—H235	119.6
H29A—C29—H29B	109.5	C235—C236—C231	119.8 (3)
N2—C29—H29C	109.5	C235—C236—H236	120.1
H29A—C29—H29C	109.5	C231—C236—H236	120.1
H29B—C29—H29C	109.5	C18—N1—C19	108.6 (3)
C112—C111—C116	118.6 (3)	C18—N1—C17	108.6 (2)
C112—C111—P1	121.9 (2)	C19—N1—C17	105.9 (2)
C116—C111—P1	119.5 (2)	C18—N1—Pd1	109.80 (18)
C113—C112—C111	120.6 (3)	C19—N1—Pd1	107.16 (19)
C113—C112—H112	119.7	C17—N1—Pd1	116.46 (18)
C111—C112—H112	119.7	C29—N2—C28	109.0 (3)
C114—C113—C112	120.4 (3)	C29—N2—C27	108.9 (2)
C114—C113—H113	119.8	C28—N2—C27	106.3 (2)
C112—C113—H113	119.8	C29—N2—Pd2	111.12 (19)
C113—C114—C115	120.0 (3)	C28—N2—Pd2	105.39 (19)
C113—C114—H114	120.0	C27—N2—Pd2	115.78 (18)
C115—C114—H114	120.0	C121—P1—C131	107.71 (14)
C116—C115—C114	120.0 (3)	C121—P1—C111	101.72 (14)
C116—C115—H115	120.0	C131—P1—C111	102.65 (14)
C114—C115—H115	120.0	C121—P1—Pd1	114.11 (10)
C115—C116—C111	120.5 (3)	C131—P1—Pd1	107.54 (10)
C115—C116—H116	119.8	C111—P1—Pd1	121.91 (10)
C111—C116—H116	119.8	C221—P2—C231	109.16 (14)
C126—C121—C122	118.8 (3)	C221—P2—C211	104.05 (14)
C126—C121—P1	120.6 (2)	C231—P2—C211	105.09 (14)
C122—C121—P1	120.6 (2)	C221—P2—Pd2	112.71 (11)
C123—C122—C121	120.6 (3)	C231—P2—Pd2	105.96 (10)
C123—C122—H122	119.7	C211—P2—Pd2	119.38 (10)
C121—C122—H122	119.7	C11—Se1—Pd1	100.25 (9)
C122—C123—C124	119.8 (3)	C21—Se2—Pd2	101.44 (9)
C122—C123—H123	120.1	N1—Pd1—P1	176.14 (7)
C124—C123—H123	120.1	N1—Pd1—Se1	92.62 (6)
C125—C124—C123	120.3 (3)	P1—Pd1—Se1	90.14 (2)
C125—C124—H124	119.9	N1—Pd1—Cl1	90.80 (7)
C123—C124—H124	119.9	P1—Pd1—Cl1	86.56 (3)
C124—C125—C126	119.9 (3)	Se1—Pd1—Cl1	175.71 (2)
C124—C125—H125	120.0	N2—Pd2—P2	173.26 (8)
C126—C125—H125	120.0	N2—Pd2—Cl2	90.79 (7)
C125—C126—C121	120.7 (3)	P2—Pd2—Cl2	86.75 (3)
C125—C126—H126	119.7	N2—Pd2—Se2	94.53 (7)
C121—C126—H126	119.7	P2—Pd2—Se2	88.69 (2)
C136—C131—C132	119.6 (3)	Cl2—Pd2—Se2	171.36 (2)
			
C16—C11—C12—C13	−2.1 (4)	C122—C121—P1—C131	117.2 (2)
Se1—C11—C12—C13	176.0 (2)	C126—C121—P1—C111	43.0 (3)
C16—C11—C12—C17	179.0 (3)	C122—C121—P1—C111	−135.3 (2)
Se1—C11—C12—C17	−2.9 (4)	C126—C121—P1—Pd1	176.2 (2)
C11—C12—C13—C14	−0.4 (5)	C122—C121—P1—Pd1	−2.1 (3)
C17—C12—C13—C14	178.4 (3)	C136—C131—P1—C121	3.9 (3)
C12—C13—C14—C15	2.4 (5)	C132—C131—P1—C121	−175.5 (2)
C13—C14—C15—C16	−1.8 (5)	C136—C131—P1—C111	−103.0 (3)
C14—C15—C16—C11	−0.8 (5)	C132—C131—P1—C111	77.6 (3)
C12—C11—C16—C15	2.7 (5)	C136—C131—P1—Pd1	127.3 (2)
Se1—C11—C16—C15	−175.4 (2)	C132—C131—P1—Pd1	−52.1 (3)
C13—C12—C17—N1	113.2 (3)	C112—C111—P1—C121	−116.8 (3)
C11—C12—C17—N1	−68.0 (4)	C116—C111—P1—C121	62.8 (3)
C26—C21—C22—C23	−0.2 (5)	C112—C111—P1—C131	−5.4 (3)
Se2—C21—C22—C23	177.3 (2)	C116—C111—P1—C131	174.2 (2)
C26—C21—C22—C27	−177.8 (3)	C112—C111—P1—Pd1	114.9 (2)
Se2—C21—C22—C27	−0.2 (4)	C116—C111—P1—Pd1	−65.6 (3)
C21—C22—C23—C24	−1.7 (5)	C226—C221—P2—C231	124.1 (3)
C27—C22—C23—C24	175.9 (3)	C222—C221—P2—C231	−59.3 (3)
C22—C23—C24—C25	1.5 (5)	C226—C221—P2—C211	−124.2 (3)
C23—C24—C25—C26	0.8 (5)	C222—C221—P2—C211	52.5 (3)
C24—C25—C26—C21	−2.8 (5)	C226—C221—P2—Pd2	6.6 (3)
C22—C21—C26—C25	2.5 (5)	C222—C221—P2—Pd2	−176.7 (2)
Se2—C21—C26—C25	−175.2 (2)	C236—C231—P2—C221	1.5 (3)
C23—C22—C27—N2	114.0 (3)	C232—C231—P2—C221	−171.0 (2)
C21—C22—C27—N2	−68.4 (4)	C236—C231—P2—C211	−109.6 (3)
C116—C111—C112—C113	0.5 (4)	C232—C231—P2—C211	77.9 (3)
P1—C111—C112—C113	−179.9 (2)	C236—C231—P2—Pd2	123.1 (2)
C111—C112—C113—C114	−0.1 (5)	C232—C231—P2—Pd2	−49.4 (3)
C112—C113—C114—C115	−0.3 (5)	C212—C211—P2—C221	−145.9 (3)
C113—C114—C115—C116	0.4 (5)	C216—C211—P2—C221	38.1 (3)
C114—C115—C116—C111	0.0 (5)	C212—C211—P2—C231	−31.2 (3)
C112—C111—C116—C115	−0.4 (4)	C216—C211—P2—C231	152.8 (2)
P1—C111—C116—C115	−180.0 (2)	C212—C211—P2—Pd2	87.4 (3)
C126—C121—C122—C123	0.6 (4)	C216—C211—P2—Pd2	−88.6 (2)
P1—C121—C122—C123	179.0 (2)	C16—C11—Se1—Pd1	−128.3 (2)
C121—C122—C123—C124	0.6 (5)	C12—C11—Se1—Pd1	53.6 (2)
C122—C123—C124—C125	−1.1 (5)	C26—C21—Se2—Pd2	−135.3 (2)
C123—C124—C125—C126	0.2 (5)	C22—C21—Se2—Pd2	47.1 (3)
C124—C125—C126—C121	1.1 (5)	C18—N1—Pd1—P1	−106.3 (10)
C122—C121—C126—C125	−1.5 (5)	C19—N1—Pd1—P1	11.5 (12)
P1—C121—C126—C125	−179.8 (2)	C17—N1—Pd1—P1	129.8 (10)
C136—C131—C132—C133	1.5 (5)	C18—N1—Pd1—Se1	117.90 (19)
P1—C131—C132—C133	−179.1 (2)	C19—N1—Pd1—Se1	−124.29 (19)
C131—C132—C133—C134	−1.7 (5)	C17—N1—Pd1—Se1	−6.0 (2)
C132—C133—C134—C135	0.7 (5)	C18—N1—Pd1—Cl1	−59.52 (19)
C133—C134—C135—C136	0.4 (5)	C19—N1—Pd1—Cl1	58.29 (19)
C132—C131—C136—C135	−0.4 (5)	C17—N1—Pd1—Cl1	176.5 (2)
P1—C131—C136—C135	−179.8 (2)	C121—P1—Pd1—N1	115.4 (11)
C134—C135—C136—C131	−0.5 (5)	C131—P1—Pd1—N1	−4.0 (11)
C216—C211—C212—C213	1.1 (5)	C111—P1—Pd1—N1	−121.9 (11)
P2—C211—C212—C213	−174.9 (2)	C121—P1—Pd1—Se1	−108.76 (10)
C211—C212—C213—C214	−0.9 (5)	C131—P1—Pd1—Se1	131.84 (11)
C212—C213—C214—C215	−0.1 (5)	C111—P1—Pd1—Se1	13.97 (12)
C213—C214—C215—C216	1.1 (5)	C121—P1—Pd1—Cl1	68.50 (11)
C214—C215—C216—C211	−0.9 (5)	C131—P1—Pd1—Cl1	−50.90 (11)
C212—C211—C216—C215	−0.2 (5)	C111—P1—Pd1—Cl1	−168.77 (13)
P2—C211—C216—C215	175.9 (2)	C11—Se1—Pd1—N1	−40.01 (12)
C226—C221—C222—C223	−2.7 (5)	C11—Se1—Pd1—P1	142.68 (10)
P2—C221—C222—C223	−179.4 (3)	C11—Se1—Pd1—Cl1	103.0 (3)
C221—C222—C223—C224	2.7 (5)	C29—N2—Pd2—P2	−127.4 (6)
C222—C223—C224—C225	−1.2 (5)	C28—N2—Pd2—P2	−9.4 (8)
C223—C224—C225—C226	−0.3 (5)	C27—N2—Pd2—P2	107.8 (6)
C222—C221—C226—C225	1.1 (5)	C29—N2—Pd2—Cl2	−58.9 (2)
P2—C221—C226—C225	177.9 (3)	C28—N2—Pd2—Cl2	59.1 (2)
C224—C225—C226—C221	0.4 (5)	C27—N2—Pd2—Cl2	176.3 (2)
C236—C231—C232—C233	0.0 (5)	C29—N2—Pd2—Se2	114.3 (2)
P2—C231—C232—C233	172.8 (2)	C28—N2—Pd2—Se2	−127.74 (19)
C231—C232—C233—C234	−2.3 (5)	C27—N2—Pd2—Se2	−10.6 (2)
C232—C233—C234—C235	2.5 (5)	C221—P2—Pd2—N2	138.4 (6)
C233—C234—C235—C236	−0.4 (5)	C231—P2—Pd2—N2	19.1 (6)
C234—C235—C236—C231	−1.9 (5)	C211—P2—Pd2—N2	−99.1 (6)
C232—C231—C236—C235	2.1 (4)	C221—P2—Pd2—Cl2	69.70 (11)
P2—C231—C236—C235	−170.4 (2)	C231—P2—Pd2—Cl2	−49.62 (11)
C12—C17—N1—C18	−59.8 (3)	C211—P2—Pd2—Cl2	−167.78 (12)
C12—C17—N1—C19	−176.3 (3)	C221—P2—Pd2—Se2	−102.97 (11)
C12—C17—N1—Pd1	64.7 (3)	C231—P2—Pd2—Se2	137.71 (11)
C22—C27—N2—C29	−59.3 (3)	C211—P2—Pd2—Se2	19.56 (12)
C22—C27—N2—C28	−176.6 (3)	C21—Se2—Pd2—N2	−34.07 (12)
C22—C27—N2—Pd2	66.7 (3)	C21—Se2—Pd2—P2	151.86 (10)
C126—C121—P1—C131	−64.5 (3)	C21—Se2—Pd2—Cl2	93.78 (19)
